# Effects of Ru and Ag cap layers on microstructure and magnetic properties of FePt ultrathin films

**DOI:** 10.1186/s11671-015-0863-x

**Published:** 2015-04-02

**Authors:** Mingfeng Liu, Tianli Jin, Liang Hao, Jiangwei Cao, Ying Wang, Dongping Wu, Jianmin Bai, Fulin Wei

**Affiliations:** Key Laboratory for Magnetism and Magnetic Materials of the Ministry of Education, Lanzhou University, Lanzhou, 730000 People’s Republic of China

**Keywords:** FePt thin film, Cap layer effect, Perpendicular magnetic anisotropy

## Abstract

The effects of Ru and Ag cap layers on the microstructure and magnetic properties of the FePt ultrathin films have been investigated. The results indicate that i) The Ag cap layer segregates from the FePt/Ag bilayer, lowers the FePt ordering temperature, promotes the FePt thin films to form island structure, and enhances the coercivity; ii) The Ru cap layer increases the FePt ordering temperature, helps to maintain smooth continuous structure film, and restrains the FePt (001) orientation and perpendicular magnetic anisotropy (PMA). The effects become more pronounced for the 3-nm-thick FePt thin films. The effects can be mainly attributed to the different melting point and thermal expansion stress between the cap layer and FePt thin films.

## Background

The *L*1_0_ phase FePt thin film is proposed to be a potential candidate for the next generation high-density perpendicular magnetic recording media for its high magnetocrystalline anisotropy (MCA) [1]. Generally, the FePt thin films deposited at room temperature are face-centered cubic (FCC) phase and show soft-magnetic behavior [2], a high annealing temperature above 550°C is needed for the ordering of face-centered tetragonal (FCT) *L*1_0_ phase FePt [3]. Several methods have been used for reducing the ordering temperature and enhancing the perpendicular magnetic anisotropy (PMA) of the *L*1_0_ phase FePt thin films, for example, i) introduction of an underlayer, ii) introduction of a cap layer, and iii) addition of a third element to form a ternary alloy compound. For the underlayer method, different underlayers have been investigated, such as Ag [4,5], Pt [6], Hf [7], Al [8], and Ti [9,10]. For the cap layer method, the Ag [11], Au [12], etc. have been introduced. The third additive elements, such as Au [13], Ag [13], Zn [14], Sn [15], and Cu [16] have been added to the FePt thin films.

A suitable cap layer is essential in magnetic recording media because it can affect the morphology, microstructure, and magnetic property of the magnetic films greatly. For example, Zhao et al. [11] found that the crystallographic ordering of the *L*1_0_ FCT FePt phase was significantly promoted when a Ag layer was deposited on top of the film; Yuan et al. [12] found that a Au cap layer was beneficial for FePt layer forming isolated grains and larger coercivity; Chen et al. [17] found that the exchange couple and magnetic reversal process can be controlled by the Cu cap layer diffusion in Cu/FePt/Pt/CrW multilayers. In this study, the effect of Ru and Ag cap layer on the microstructure and magnetic properties of ultrathin FePt films was investigated by using X-ray diffraction (XRD), scanning electron microscope (SEM), vibrate sample magnetometer (VSM), and anomalous Hall effect (AHE). We found that the Ag cap layer segregates from the FePt/Ag bilayer after annealing, which lowers the FePt ordering temperature, promotes the FePt thin films to form island structure, and enhances the coercivity, whereas the Ru cap layer shows a completely inverse behavior. The effects can be mainly attributed to the different melting point and thermal expansion stress between the cap layer and the FePt thin films.

## Methods

The FePt (*t* nm, *t* = 3, 10) single layers and FePt (*t* nm, *t* = 3, 10)/X (5 nm, X = Ru, Ag) bilayers were deposited on the thermally oxided Si (100) substrates by Fe and Pt magnetron co-sputtering using a Kurt Lesker CMS-18 sputtering system (Kurt J. Lesker Company, Jefferson Hills, USA) at room temperature. The chamber pressure of the sputtering system was less than 1 × 10^−7^ Torr, and the deposition gas was 5 mTorr high purity Ar gas. After the deposition, the samples were annealed in vacuum for an hour with a base pressure less than 1.2 × 10^−5^ Torr. The structure of the samples was characterized by Rigaku D/Max-2000 XRD (Rigaku, Tokyo, Japan) with Cu K_α_ radiation. The ordering parameters of FePt phase were calculated by following equation: *S*^2^ = (*I*_001_/*I*_002_)_meas_/(*I*_001_/*I*_002_)_calc_ [18,19], where *I*_001_ and *I*_002_ are the integrated intensity of (001) superlattice and (002) fundamental diffractions, respectively. The composition of the FePt layer is Fe_59_Pt_41_, confirmed by inductively coupled plasma atomic emission spectroscopy (ICP-AES) method. The morphology of the samples was obtained by Hitachi S4800 field emission SEM (Hitachi, Tokyo, Japan). The magnetic property was characterized by TOEI VSM-5S VSM (Toei Industry Co., Ltd., Tokyo, Japan) at room temperature. The perpendicular magnetic hysteresis loops of FePt (3 nm)/X (5 nm) ultrathin films were characterized by a home-built AHE measurement system, because of the limited accuracy of the VSM for the FePt ultrathin films.

## Results

**Figure 1:**
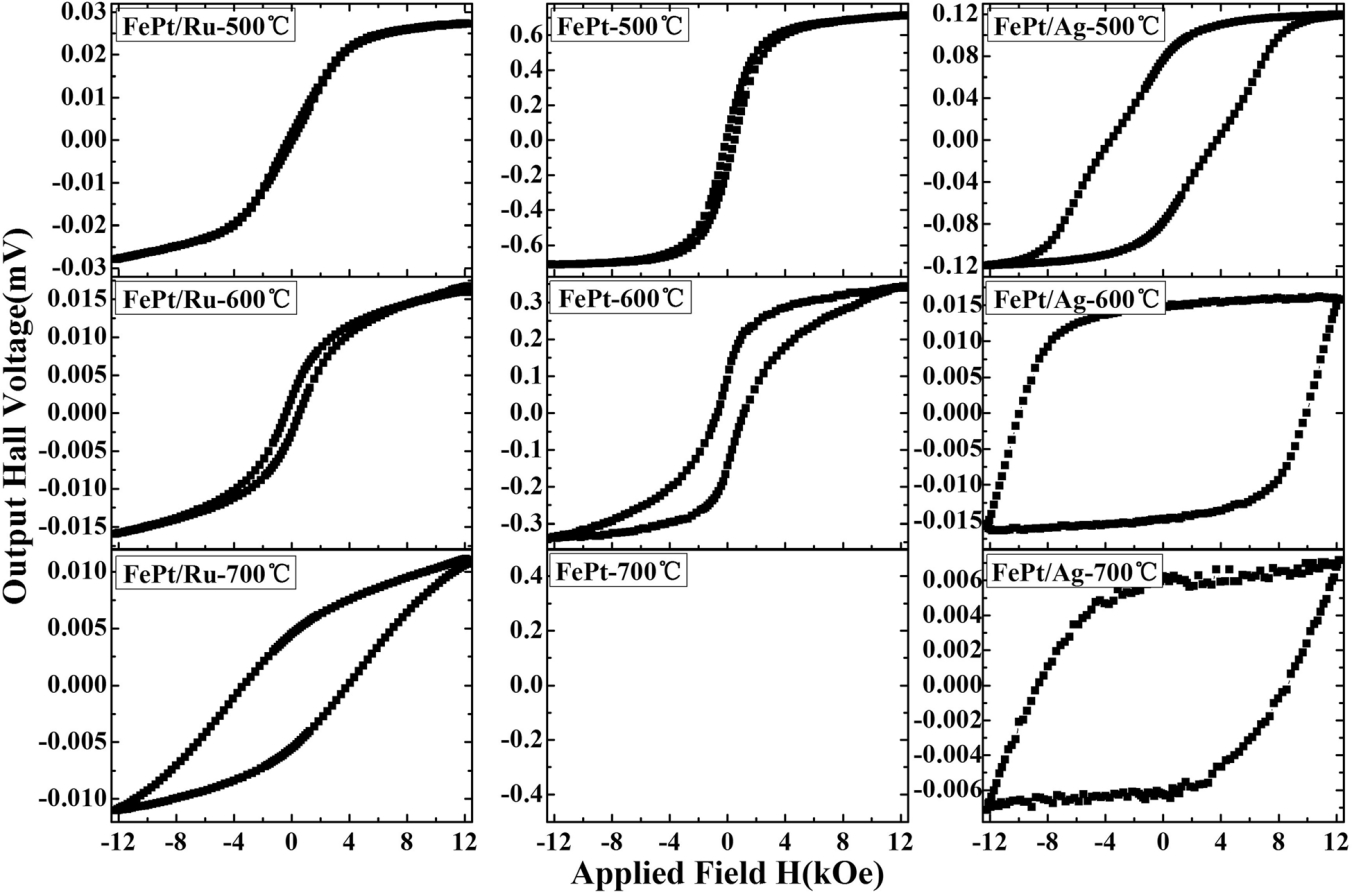
**AHE hysteresis loops of FePt (3 nm) thin films.** AHE hysteresis loops of FePt (3 nm) thin films with Ru and Ag and without a cap layer annealed at 500°C, 600°C, and 700°C.

**Figure 2:**
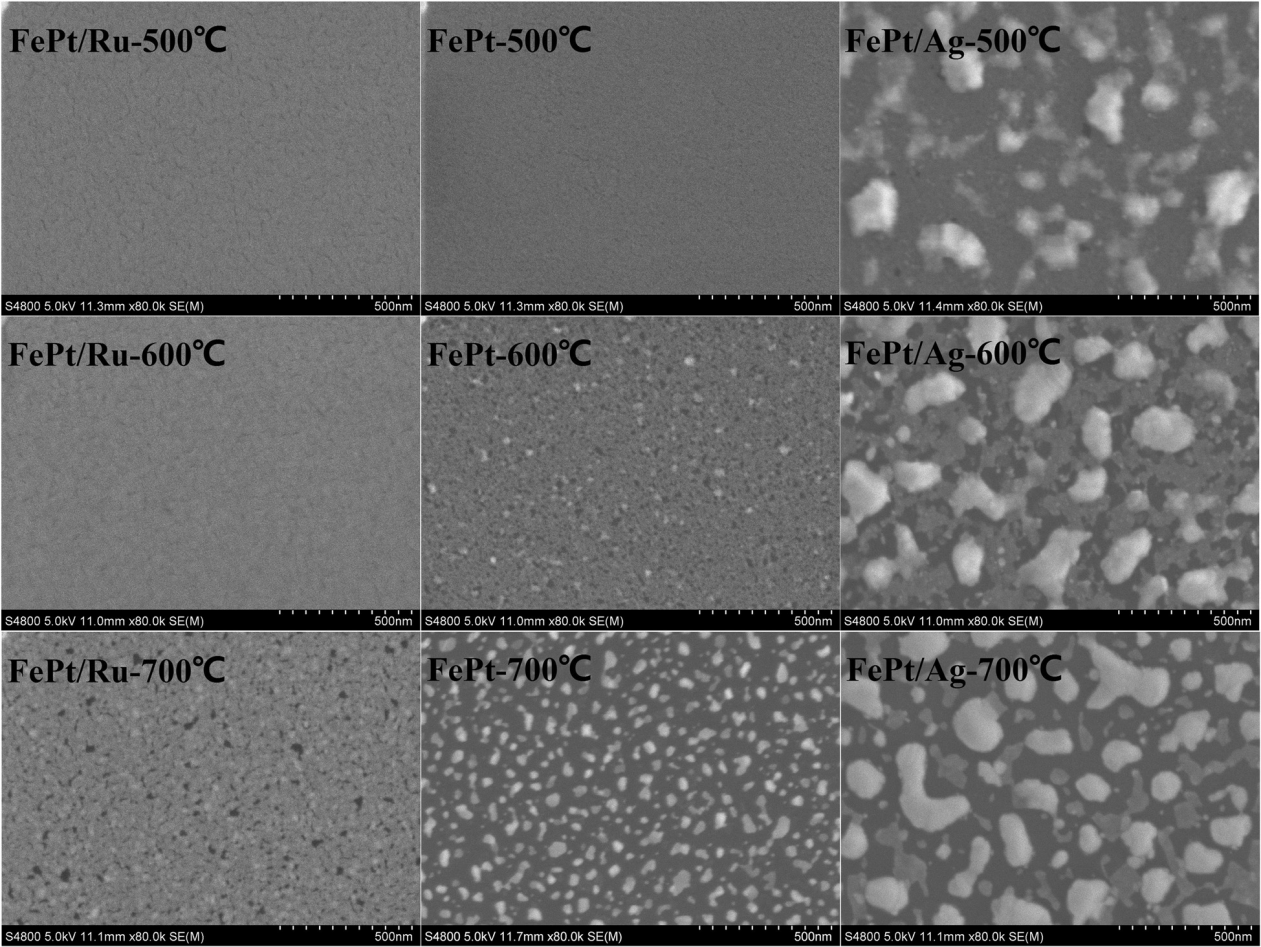
**SEM images of the FePt (3 nm) thin films.** SEM images of the FePt (3 nm) thin films with Ru and Ag and without a cap layer annealed at 500°C, 600°C, and 700°C.

Figure 1 shows the AHE loops of FePt (3 nm) thin films with Ru, Ag, and without a (FePt single layer) cap layer, annealed at different temperatures. Since some samples were electrically isolated after annealing, to improve the conductivity, a 5-nm Pt top layer was deposited on the annealed films for the AHE testing at room temperature. For the films annealed at 500°C, the FePt/Ru shows nearly zero coercivity, and the FePt single layer shows a very low perpendicular coercivity, while the FePt/Ag film shows hard magnetic properties with a dramatically high coercivity of 4 kOe, which is obviously larger than that of the FePt/Ru and FePt single layer. This may arise from the ordering of FePt (3 nm) thin films at a lower temperature below 500°C in FePt/Ag bilayer. At 600°C, a more or less coercivity increase can be observed in all the films, among them, the FePt/Ag shows a large coercivity up to 10.1 kOe and good squareness, even though the film is not saturated by the 12-kOe field. At 700°C, the coercivity of the FePt/Ru increases obviously, and the FePt/Ag is more difficult to be saturated and only a minor loop is obtained. For the FePt single layer annealed at 700°C, no obvious hall voltage was measured (not shown) even though a 5 nm Pt top layer was deposited after annealing. That may be due to the completely isolated particle microstructure of the thin films (as shown in Figure 2); as a result, the current scarcely flows through the FePt particles. Note that the coercivity of the Ru capped films and that of films without a cap layer are much lower than that with Ag, partially because of the ordering temperature and microstructure of two kinds of films, which will be discussed in detail later.

**Figure 3:**
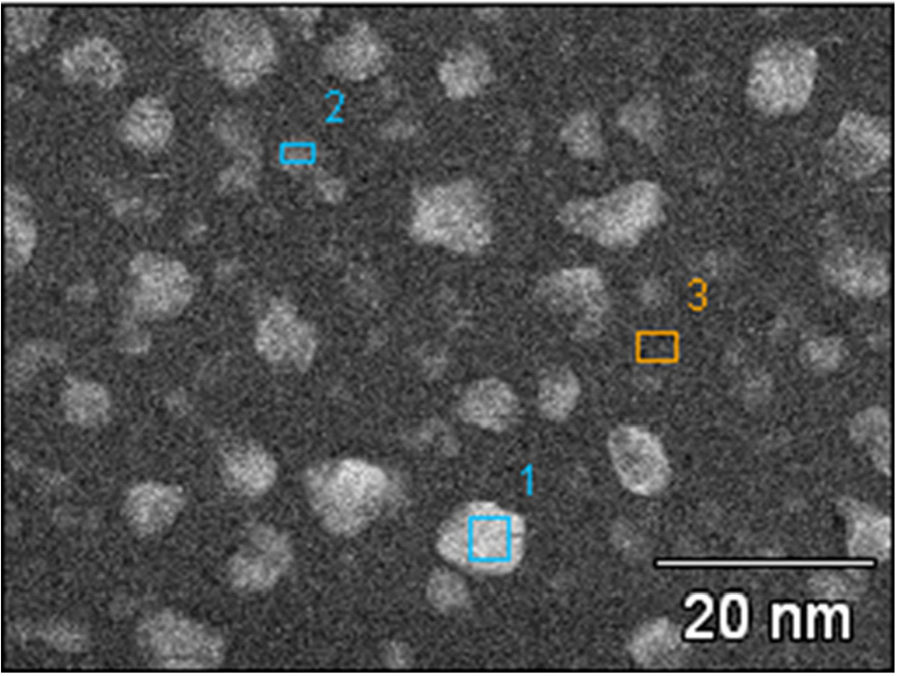
**EDS micro-area element analysis SEM image of FePt (3 nm)/Ag (5 nm) annealed at 500°C.** The Fe/Pt/Ag atom ratios 1: 3.67/0/96.33, 2: 18.46/0/81.54, 3:41.59/41/17.41 in areas 1, 2, and 3, respectively.

Figure 2 displays the SEM images of the FePt (3 nm) thin films with Ru and Ag and without a cap layer, annealed at different temperatures. For the films annealed at 500°C, the FePt/Ru and FePt single layers show a perfectly continuous film; whereas large isolated particles are found in FePt/Ag bilayers. To analyze the microstructure of the FePt/Ag films, a micro-area element analysis has been carried out by using electronic dispersive spectrometer (EDS); as shown in Figure 3, the Fe/Pt/Ag atom ratios in areas 1, 2, and 3 are 3.67/0/96.33, 18.46/0/81.54, and 41.59/41/17.41, respectively. This suggests that in the 500°C-annealed FePt/Ag bilayer, the Ag layer becomes a discontinuous island structure. When the annealing temperature is increased to 600°C, the Ru capped film surface keeps being continuous, while both FePt layer and Ag layer become discontinuous in the FePt/Ag bilayer and show a typical granular Ag island and a partially isolated schistose structure in the FePt layer. The FePt single layer becomes obviously granular in morphology with massive holes. At 700°C, a granular structure with hole morphology is observed for the FePt films with the Ru cap layer. The FePt single film shows a completely isolated, homogeneous, particle structure; the Ag islands further grow up and the schistose structure in the FePt layer becomes completely isolated in the FePt/Ag films.

**Figure 4:**
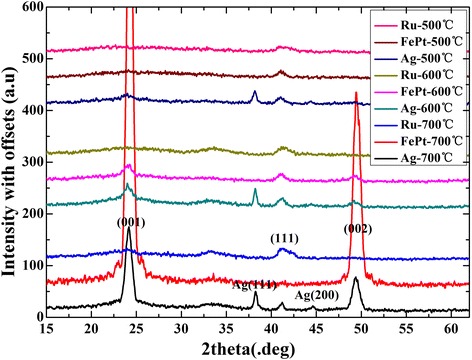
**XRD patterns of FePt (10 nm) thin films.** XRD patterns of FePt (10 nm) thin films with Ru and Ag and without a cap layer annealed at 500°C, 600°C, and 700°C.

The above results suggest that Ru and Ag cap layers significantly affect the microstructure and magnetic properties of 3-nm-thick FePt films effectively. The Ru cap layer helps to maintain the film surface smooth and decrease the perpendicular coercivity, while the Ag layer is favorable to form particle structure and higher perpendicular coercivity at a low annealing temperature. However, the mechanism of the effects is still not well understood because the crystal structure and magnetic anisotropy is not easy to be measured in the FePt (3 nm) ultrathin films. To further investigate the effects of cap layers on thicker FePt thin films, 10-nm-thick FePt thin films with Ru and Ag and without cap layers were deposited and studied. Figure 4 shows the XRD patterns of them annealed at different temperatures. For the films annealed at 500°C, a weak FePt (001) peak is observed in the film with the Ag cap layer, while no FePt (001) peak appears in the films with Ru and without a cap layer. At 600°C, the (001) peak starts to appear in the FePt single layer, and the Ag capped FePt thin film shows a stronger FePt (001) peak, while no obvious FePt (001) peak appears in the films with the Ru cap layer. At 700°C, the FePt (001) peak becomes stronger in the FePt/Ag and FePt single layer, while no FePt (001) peak is observed for the FePt/Ru bilayer. These results indicate that the Ag cap layer helps to form the FePt (001) phase at a lower temperature of 500°C, while the Ru cap layer restrains the ordering process with (001) orientation until 700°C.

**Figure 5:**
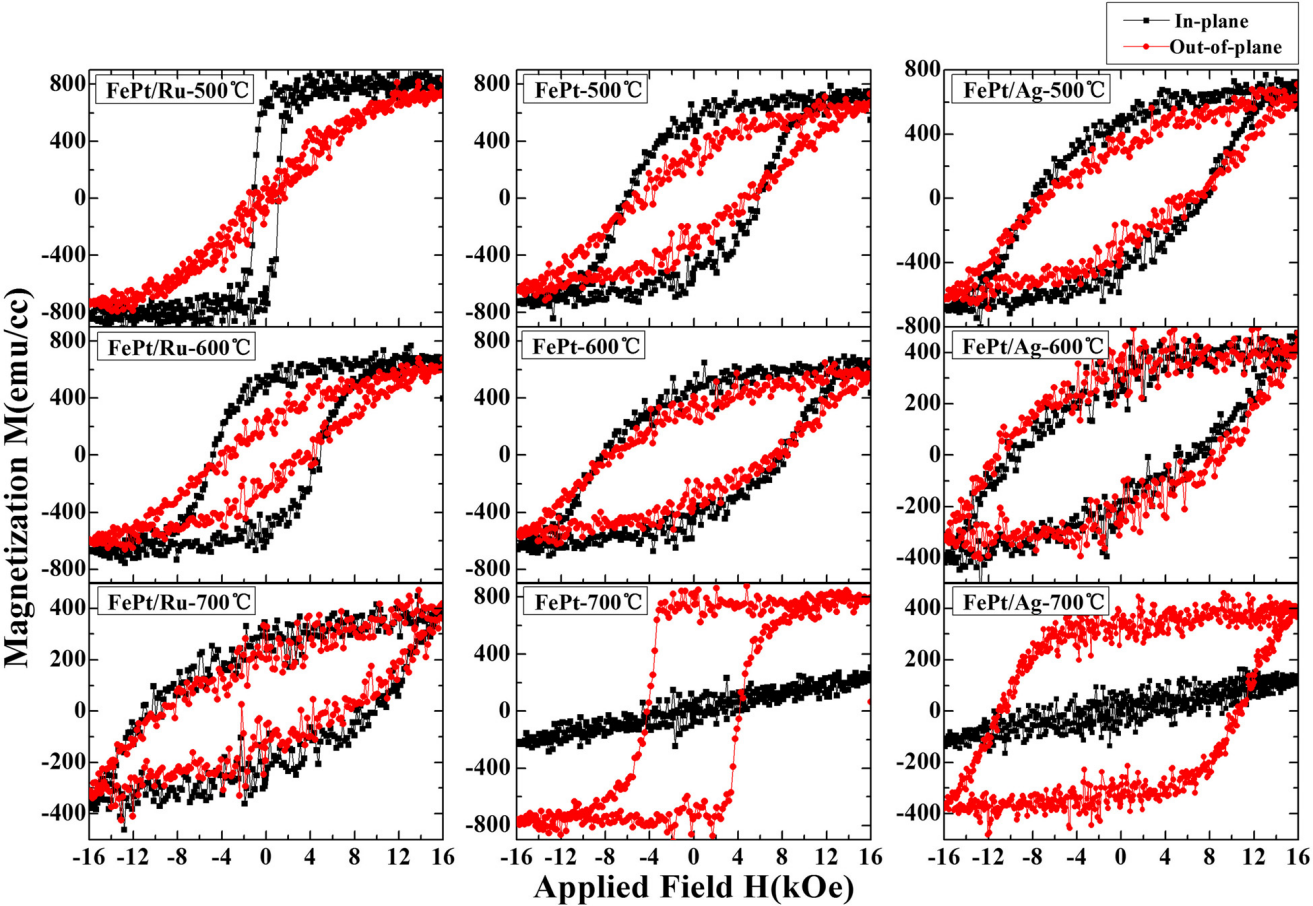
**Magnetic hysteresis loops of FePt (10 nm) thin films.** Magnetic hysteresis loops of FePt (10 nm) thin films with Ru and Ag and without a cap layer annealed at 500°C, 600°C, and 700°C.

Figure 5 shows the magnetic hysteresis loops of FePt (10 nm) thin films with different cap layers. For the films annealed at 500°C, the FePt/Ru bilayer shows low out-of-plane coercivity and in-plane easy axis, resulting from the large demagnetization field along out-of-plane direction and low ordering parameter. Compared to the FePt/Ru bilayer, the FePt single layer and FePt/Ag show large coercivity and almost same value for both in- and out-of-plane directions, because of higher ordering and the dominant (111) orientation, as shown in Figure 4. At 600°C, for the FePt/Ru bilayer, the coercivity increases more observably, suggesting that the ordering process occurs mostly at 500°C ~ 600°C. These indicate that the FePt single layer and FePt/Ag have a lower ordering temperature below 500°C, while the Ru cap layer restrains the FePt ordering process. For the films annealed at 700°C, the FePt single layer and FePt/Ag bilayer show a clear PMA, indicating that the easy axis of the FePt films turn to out-of-plane. It should be noted that for all the films capped with Ag layer, the hysteresis loops in both directions are not saturated at 16 kOe. The intrinsic Hc value should be larger than that shown in Figure 5. In addition, the film without a cap layer shows better rectangle hysteresis and lower perpendicular coercivity than that of FePt/Ag, although almost an equivalent ordering parameter was obtained for the FePt single layer and FePt/Ag bilayer. The discrepancy in the coercivity values may result from the different magnetization reversal process: the FePt single layer shows a typical domain wall movement magnetization behavior, while the FePt/Ag shows a typical coherent rotation magnetization behavior. This will be discussed in detail in the next paragraph after the illustration of the SEM images of the films.

**Figure 6:**
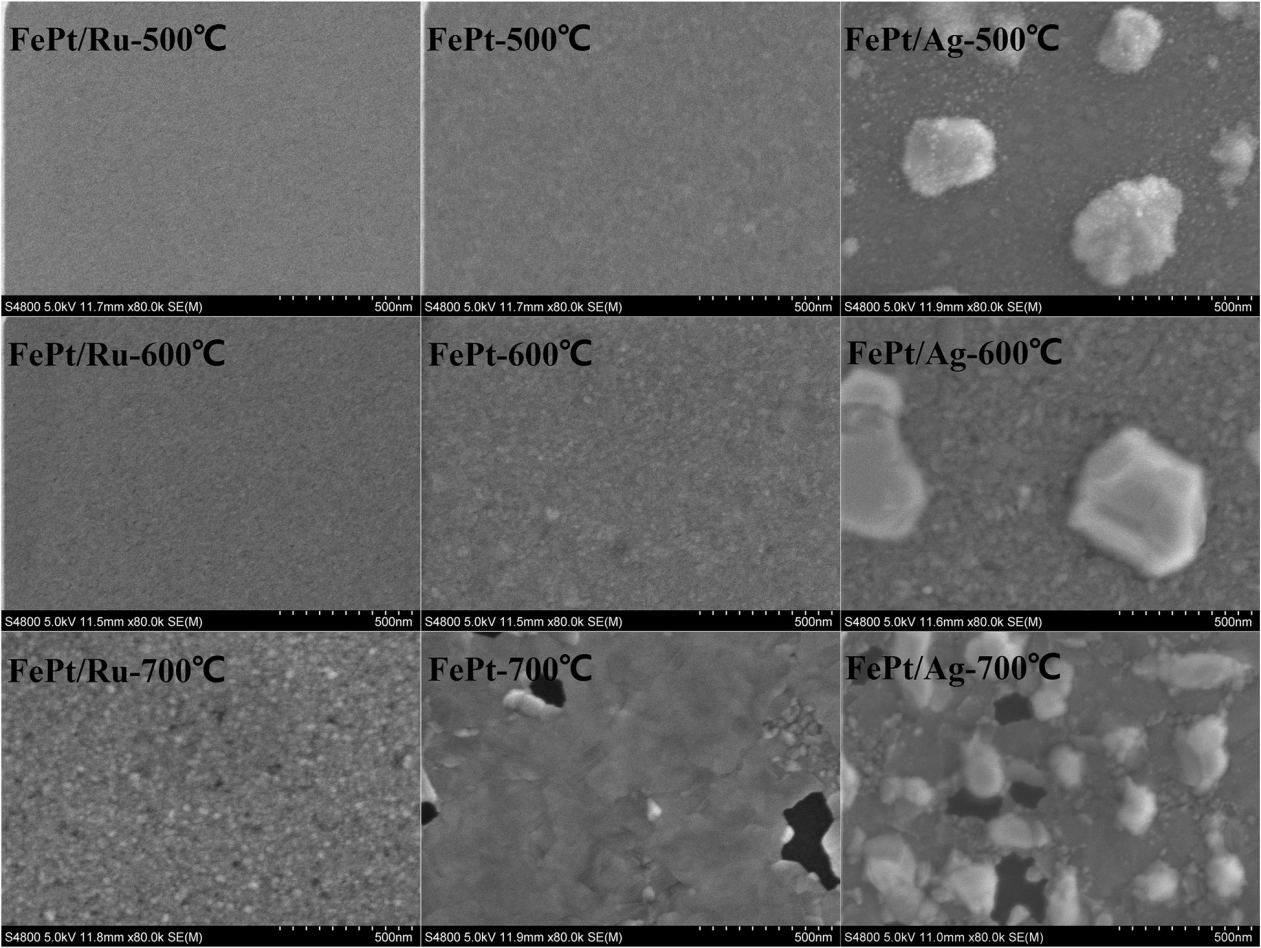
**SEM images of FePt (10 nm) thin films.** SEM images of FePt (10 nm) thin films with Ru and Ag and without a cap layer annealed at 500°C, 600°C, and 700°C.

Figure 6 displays the SEM images of annealed FePt (10 nm) thin films with different cap layers. For the films annealed at 500°C, the FePt/Ru and FePt single layers show perfectly continuous films, while FePt/Ag film shows a structure of Ag islands lying on the continuous FePt layer. When the annealing temperature is increased to 600°C, the FePt/Ru bilayer keeps being continuous, while small particles of the FePt underlay can be observed. The FePt single layer keeps being continuous and becomes obviously rough. The FePt/Ag bilayer consists of typical white islands (Ag) and continuous granular underlayer (FePt). At 700°C, the granular structure with holes can be observed in the FePt/Ru bilayer, and the FePt single layer shows a continuous schistose structure with shrinkage holes, while the FePt/Ag bilayer shows Ag islands and a more isolated granular structure in the FePt layer. Based on the above XRD and SEM results, for the 700°C-annealed FePt single layer and FePt/Ag bilayer, the difference of the coercivity can be understood: for the FePt/Ag bilayer, it shows a mixture texture of (001) and (111). As we know, the boundaries among the grains with different orientation may act as pinning sites, which impede the domain wall movement, resulting in a typical coherent rotation magnetization behavior and high coercivity; while for the FePt single layer, the film consists of a μm-sized schistose structure with dominant (001) texture, no grain boundary acts as pinning site, and the domain wall movement may happen, resulting in a typical domain wall movement behavior and small coercivity.

## Discussion

According to the above results, we can find that different cap layers show significant different effects on the FePt film, including the structure, ordering temperature, orientation, surface morphology, and magnetic properties. The different morphology in the annealed bilayers can be attributed to the surface energy and melting point of the cap layers. Surface energy and melting point are important factors that influence various surface phenomena including faceting, roughening, crystal growth, catalytic behavior, and surface segregation during annealing [20]. Such as the FePt/Ag bilayer, at 500°C, the Ag cap layer segregates from the FePt/Ag bilayer and forms Ag(111) and Ag(200) islands, while the FePt layer maintains continuous smooth surface, which can be attributed to the low melting point (961°C) of the metal Ag and the high melting point of FePt (>1,519°C) [2]. For the FePt/Ru bilayer, because of a high melting point and surface energy of Ru, the films keep being continuous after 600°C annealing.

The thermal expansion stress (thermal stress) arising from the different coefficients of thermal expansion (CTE) can be much more important for the ordering enhancement in the FePt/Ag bilayer film. The thermal stress arising from the difference in CTE can be calculated [21]:

$$ \sigma =\varDelta \alpha \varDelta TE/\left(1-\mu \right) $$

where ∆*α* is the difference in thermal expansion coefficient between the cap layer and FePt film, ∆*T* is the change in temperature between room temperature and the annealing temperature, *E* is the elastic modulus of the FePt film, 180 GPa, and *μ* is Poisson’s ration 0.33. A negative value of *σ* means that the film is in compression, while a positive value means it is in tension. The calculated thermal stress between the different cap layers and FePt film has been listed in Table 1. We note that the Ag cap layer produces a positive thermal stress of 1.17 GPa on the FePt layer at 500°C, while the Ru cap layer has a negative value of −0.54 GPa. For the Ag capped FePt film, the in-plane tension thermal stress, originating from the Ag cap layer, would force the FePt thin film in-plane unit cell expansion, which would relieve the externally applied stress, resulting in (001) orientation in the FePt films. Additionally, considering that the thermal stress originates from the SiO_2_ substrates of −1.34 GPa and the intrinsic residual tensile stress in sputtering thin metal films of 1.2 to 1.5 GPa [21,22], the stress originating from the substrates will be negligible. Thus, the total thermal stress is equal to that originating from the cap layer.

**Table 1:** **The melting point and CTE stress of the FePt layer and the cap layer**

**Bilayer**	**Cap layer material**	**Melting point (°C)**	**CTE (ppm/K)**	**Thermal stress [** 21 **] (GPa) at 500°C**	**References**
FePt/Ru	Ru	2,250	<6.5	−0.54	[23]
FePt/Ag	Ag	961	19.2	1.17	[4,24]
FePt	Without	>1,519	10.5	0	[21]
Substrate	SiO_2_	1,670	0.5	−1.34	[25]

On the contrary, the Ru cap layer shows a completely inverse behavior compared to the Ag cap layer. This can be attributed to the higher melting point (2,250°C) than the FePt film, and the negative thermal stress −0.54 GPa on FePt thin films listed in the Table 1. Based on the discussion above, the high melting point of Ru helps the Ru cap layer maintain a smooth continuous film until the ordered FePt particles are formed. And the negative thermal stress of the cap layer generates a total in-plane compression stress on the FePt film, which restrains the FePt ordering process. Lastly, we note that the effects of the cap layer on magnetism of 3-nm-thick FePt films are much more pronounced than the 10-nm-thick films under the same annealing temperatures.

## Conclusions

In summary, the melting point and thermal expansion stress originating from different CTE plays an important role on the FePt/X (5 nm, X = Ag, Ru) bilayer annealing process. The Ag cap layer segregates from the FePt/Ag bilayer, lowers the FePt ordering temperature, promotes the FePt thin films to form an island structure, and enhances the coercivity. The Ru cap layer increasing the FePt ordering temperature helps to maintain smooth continuous surface, restrains the FePt (001) orientation and the perpendicular magnetic anisotropy. The effects become much more pronounced for the 3-nm-thick FePt thin films. The effects can be mainly attributed to the low melting point and positive thermal expansion stress originating from the Ag cap layer and the high melting point and negative thermal expansion stress originating from the Ru cap layer.
